# 1997. Risk Factors and Outcomes of *Mycobacterium abscessus* Complex (MABC) Acquisition after Lung Transplantation

**DOI:** 10.1093/ofid/ofad500.124

**Published:** 2023-11-27

**Authors:** Sophie E Nick, Michael E Yarrington, John M Reynolds, Deverick J Anderson, Arthur W Baker

**Affiliations:** Duke University School of Medicine, Durham, NC; Duke University Health System, Durham, North Carolina; Duke University School of Medicine, Durham, NC; Duke Center for Antimicrobial Stewardship and Infection Prevention, Durham, North Carolina; Duke University School of Medicine, Durham, NC

## Abstract

**Background:**

Lung transplant recipients are at increased risk of MABC acquisition. The objective of this study was to analyze risk factors and outcomes of early post-transplant MABC acquisition.

**Methods:**

We conducted a single center retrospective case-control study of patients who underwent lung transplant from 1/1/2012 – 12/31/2021. Cases were patients with *de novo* MABC acquisition within 90 days post-transplant. Controls had no positive MABC cultures and were matched 1:3 with cases based on age and transplant date. Recipient demographics, pre-/peri-operative characteristics, and outcomes for 1-year post-transplant were analyzed.

Conditional logistic regression was used to determine independent risk factors for MABC acquisition. The reference model included all variables with a *P* value ≤ 0.2 in univariate analysis after accounting for collinearity and epidemiologic plausibility. Confounding variables and variables with an adjusted *P* value ≤ 0.05 were included in the final model.

**Results:**

79 cases and 237 controls were identified out of 1,145 lung transplants. The median time to MABC isolation was 33 days (IQR 11 – 59) after transplant. The first positive culture was pulmonary for 70 (89%) cases; 59 (75%) cases were treated with antibiotics targeting MABC.

Univariate analysis found cases were more likely than controls to have CMV mismatch (D+/R-), bilateral transplant, higher end-match allocation score, increased ventilator time pre- and post-transplant, and days in the hospital before MABC index date (Table 1). IPF diagnosis and receipt of azithromycin were protective. Multivariable analysis showed only post-transplant ventilation for ≥ 2 days was independently associated with MABC (Table 2).

Compared to controls, cases required more days of hospitalization after the MABC index date (28 vs. 12 days; p=0.01) and had decreased 1-year post-transplant survival (78% vs. 89%; p=0.02) (Figure 1). When stratified by subspecies, 1-year survival was 69% for *M. abscessus* and 89% for *M. massiliense* (p=0.10).

Table 1.

**Figure:**
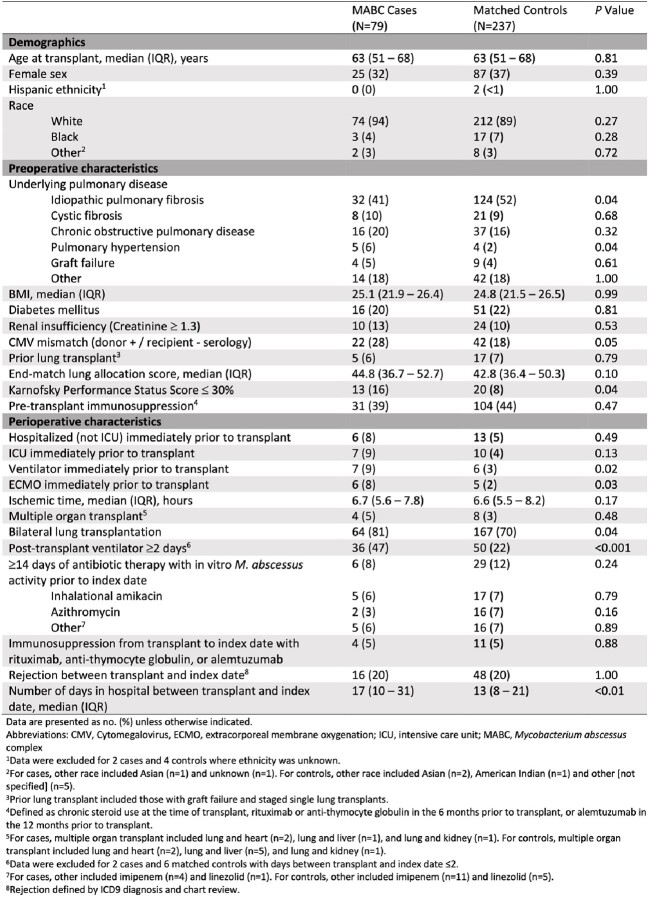

Lung transplant recipient characteristics

Table 2.

**Figure:**
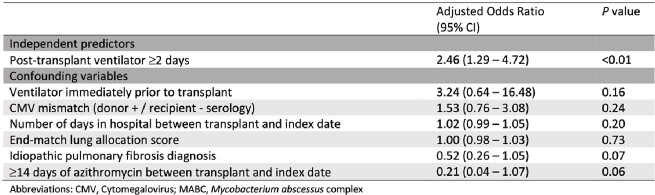

Conditional logistic regression model constructed via backward elimination to determine independent predictors and confounders of MABC acquisition

Figure 1.

**Figure:**
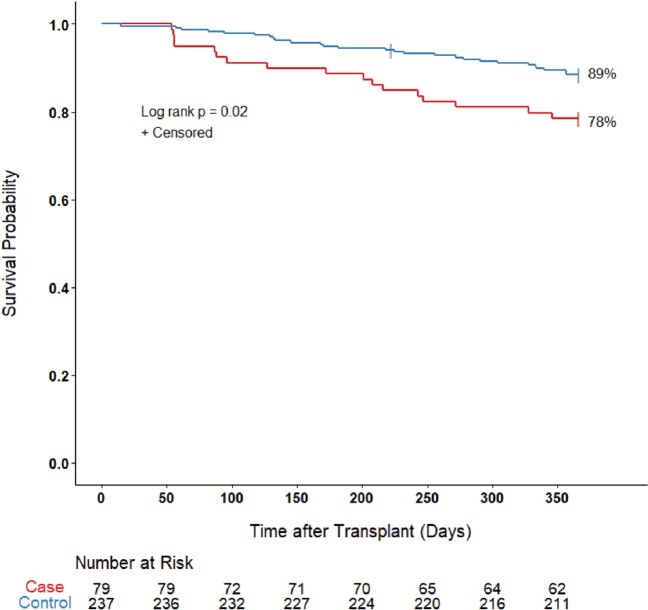

Kaplan-Meier survival curve comparing cases and controls for 1-year after transplant

**Conclusion:**

In this large case-control study, post-transplant ventilator duration was associated with MABC acquisition, which in turn was associated with increased hospital days and mortality. Further studies are needed to determine the best strategies for MABC prevention and surveillance.

**Disclosures:**

**Arthur W. Baker, MD, MPH**, Insmed: Grant/Research Support|Medincell: Advisor/Consultant

